# Voices From the NICU: Exploring Lived Maternal Experiences and Challenges in Caring for Preterm Newborns in Central Ethiopia: A Phenomenological Study

**DOI:** 10.1002/hsr2.72321

**Published:** 2026-04-19

**Authors:** Melaku Eriso, Markos Selamu, Bisrat Feleke Bubamo, Gizachew Beykaso, Hamdela Tumiso, Eyob Ketema Bogale, Genanew Kassie Getahun, Feleke Doyore Agide

**Affiliations:** ^1^ School of Public Health College of Medicine and Health Sciences, Wachemo University Hossana Ethiopia; ^2^ Department of Family Health Hossana College of Health Science Hossana Ethiopia; ^3^ Health Promotion and Behavioral Science Department, School of Public Health College of Medicine and Health Science, Bahir Dar University Bahir Dar Ethiopia; ^4^ Menelik II Medical and Health Science College Addis Ababa Ethiopia; ^5^ Department of Public Health, School of Allied Health Sciences Kampala International University Kampala Uganda

**Keywords:** Mother's experience, neonatal intensive care unit, preterm newborn

## Abstract

**Background and Aims:**

Neonatal intensive care units are important to save the lives of premature babies. However, the mothers face several stressful situations during the period of admission. The aim of this study was therefore to explore the experience of mothers with premature babies in the neonatal intensive care unit of the Nigist Elleni Mohammed Memorial Comprehensive Specialized Hospital in Hossana, Central Ethiopia.

**Methods:**

A phenomenological‐descriptive study was conducted among thirteen mothers who were purposefully selected. Data were coded and transcribed verbatim, translated, and inductive thematic analysis was employed. ATLAS.ti8 software was used to manage data. The findings were presented systematically by stating each major theme and category with the support of the evidence of direct quotes.

**Results:**

This study explored the experiences of mothers with preterm newborns in a neonatal intensive care unit. Five major themes and related categories included mothers’ emotional experiences, maternal expectations, healthcare professionals’ needs, factors related to healthcare facilities, and perceived incompetence in the care of premature infants. The study found that mothers experienced negative emotions due to medication shortages, inadequate laboratory tests, a lack of toilets, inadequate water supplies, and a lack of confidence in caring for their infants, despite a range of support from nursing staff, as an important public health issue.

**Conclusion:**

This study revealed that mothers of preterm infants in NICUs face significant emotional and practical challenges. Key issues include inadequate medical resources, poor facility conditions, and low maternal confidence. Addressing these gaps is crucial for improving neonatal and maternal care.

## 1 Introduction

Globally, an estimated 15 million infants are born preterm each year, contributing substantially to neonatal deaths and long‐term developmental complications. Preterm birth, defined as delivery before 37 completed weeks of gestation, remains a major global public health challenge and a leading cause of neonatal mortality and long‐term morbidity. The burden of preterm birth is disproportionately concentrated in low‐ and middle‐income countries (LMICs), where health systems often face limitations in providing specialized neonatal care. In 2015, approximately 20.5 million preterm births were reported worldwide, and nearly 91% occurred in LMICs, particularly in South Asia (48%) and Sub‐Saharan Africa (24%) [1, 2, 3]. These patterns highlight persistent inequalities in maternal and newborn health outcomes and emphasize the need for context‐specific strategies to address the consequences of prematurity.

Ethiopia is among the countries where preterm birth continues to contribute substantially to neonatal morbidity and mortality. Evidence indicates that the pooled prevalence of preterm birth in Ethiopia is approximately 10.48% [4, 5]. Preterm birth is estimated to account for nearly one‐third of neonatal deaths annually [6, 7, 8], making it one of the most important contributors to neonatal mortality in the country. In addition to its direct impact on newborn survival, preterm birth is often associated with maternal health complications such as hypertensive disorders of pregnancy, infections, and obstetric emergencies that threaten both maternal and neonatal well‐being [9, 10, 11]. Addressing preterm birth is therefore critical for improving outcomes across the continuum of maternal and newborn health.

The causes of preterm birth are complex and multifactorial. Some preterm deliveries occur spontaneously due to premature labor or premature rupture of membranes, often linked to infections or inflammatory processes. In other cases, preterm birth is medically indicated due to maternal or fetal complications such as pre‐eclampsia, fetal growth restriction, or multiple pregnancies [12, 13, 14]. In many resource‐limited settings, however, accurate documentation and classification of preterm births remain challenging because of incomplete birth registration and limited clinical records. These gaps hinder a comprehensive understanding of the determinants and consequences of preterm birth, particularly in LMIC contexts.

While considerable attention has been given to the clinical management of premature infants, less emphasis has been placed on the experiences of mothers whose newborns require intensive care. In many hospital settings, mothers may initially have limited physical contact with their newborns due to infection prevention measures, the infant's medical instability, or structural constraints within the NICU [4, 15]. Such separation may disrupt early mother–infant bonding and lead to feelings of anxiety, helplessness, and uncertainty. The NICU environment can further intensify maternal stress. Mothers are frequently exposed to unfamiliar medical technologies, continuous monitoring devices, alarms, and specialized clinical procedures. At the same time, concerns about the infant's survival and potential long‐term complications may create significant emotional distress [16, 17]. Preterm birth can also interrupt mothers’ expected transition to parenthood, affecting their physical, psychological, and social preparation for motherhood. Instead of experiencing a typical postpartum period, many mothers must cope with fear, uncertainty, and a perceived loss of their parental role during their infant's hospitalization [4, 13, 18]. These challenges may ultimately affect maternal well‐being, early bonding, and the caregiving relationship between mother and infant [19].

Providing adequate emotional, informational, and social support to mothers during NICU hospitalization is therefore essential. Evidence suggests that insufficient support may negatively influence maternal psychological well‐being and the development of healthy mother–infant attachment [20]. However, neonatal care in many settings remains primarily focused on the clinical stabilization of the infant, with comparatively limited attention given to the emotional and experiential needs of parents. Previous studies also indicate that parental experiences in NICU settings vary depending on sociocultural context, healthcare infrastructure, communication with healthcare providers, and opportunities for parental involvement in neonatal care [21, 22, 23, 24].

Despite increasing recognition of the importance of family‐centered neonatal care, limited qualitative evidence exists on the lived experiences of mothers with preterm infants in NICU settings in Ethiopia. Most existing studies focus primarily on clinical outcomes rather than the psychosocial experiences of mothers. Therefore, this study aims to explore the experiences of mothers with premature infants admitted to the neonatal intensive care unit at Wachemo University Nigist Elleni Mohammed Memorial Comprehensive Specialized Hospital in Hossana, Central Ethiopia. Understanding these experiences will provide important insights to guide improvements in maternal support systems and the implementation of family‐centered care in neonatal intensive care settings.

## 2 Methods

### 2.1 Study Setting

The study was carried out at Wachemo University Nigist Elleni Mohammed Memorial Comprehensive Specialized Hospital (WCUNEMMCSH) in Hossana town, Hadiya Zone, Central Ethiopia, located approximately 232 km in the Southern part of Addis Ababa. WCUNEMMCSH is a public referral hospital providing comprehensive medical services, including surgical, obstetric and gynecological, and pediatric care. The hospital serves a large catchment population from Hossana and the surrounding districts. Due to high patient inflow and limited alternative healthcare facilities in the region, the hospital frequently operates beyond its intended capacity, particularly in the maternity ward and NICU. As a result, shortages of beds, essential medical equipment, and trained healthcare professionals such as neonatal nurses, pediatricians, and midwives are common. In the NICU, a single nurse may care for six to ten neonates per shift, which may affect timely care and increase the workload of healthcare providers. In addition to these resource constraints, sociocultural factors in the Hadiya Zone influence maternal and newborn healthcare utilization. Traditional beliefs related to childbirth and neonatal illness, reliance on home remedies, and limited male involvement in facility‐based care may contribute to delays in seeking medical attention and may influence the acceptance of recommended neonatal care practices, including Kangaroo Mother Care.

### 2.2 Study Design and Period

A phenomenological study was conducted from September 21, 2023, to February 15, 2024.

### 2.3 Participants and Sampling Strategy

This study aimed to explore mothers’ experiences of having a preterm infant admitted to the neonatal intensive care unit (NICU). A purposive sampling technique was used to recruit 13 mothers whose preterm newborns had been admitted to the NICU at Wachemo University Nigist Elleni Mohammed Memorial Comprehensive Specialized Hospital (WCUNEMMCSH) for at least 1 week. The researchers reviewed the length of stay of the newborns and identified mothers who were willing to share their experiences through in‐depth interviews. The number of participants was determined based on data saturation, whereby no new information or themes emerged, and participants’ responses became repetitive. Potential participants were informed about the purpose and procedures of the study, with emphasis on the voluntary nature of participation and the assurance of privacy and confidentiality. Mothers who agreed to participate were then invited to schedule a convenient date and time for the interview.

### 2.4 Inclusion and Exclusion Criteria

Participants were eligible for inclusion if they had a preterm infant (born before 37 weeks of gestation) admitted to the NICU during the study period; if they had stayed with or visited their infant in the NICU for at least 48 h, allowing them to gain some experience with the NICU environment; and were willing to share their experiences and able to provide informed consent are included in the study. However, mothers were critically ill or medically unstable at the time of data collection; experienced severe emotional distress that could make participation harmful; had infants who were transferred to another facility immediately after birth without NICU admission, and who were unable to participate in an in‐depth interview due to communication barriers or other limitations were excluded from the study.

### 2.5 Data Collection

The study used In‐depth interviews (IDI) to gather data on mothers’ experiences. A semi‐structured interview guide was developed based on the study objectives and literature review. The guide was prepared in English and translated into the local language, and then translated back to English by another person to maintain consistency. Face‐to‐face interviews with mothers were conducted in a quiet room adjacent to the morning session, utilizing a tape recorder and concurrent field note‐taking. The guide addressed diverse topics and included probes and follow‐up questions for deep insight. The interviews lasted 30 to 60 min, and data collection continued until data saturation. The data was collected by researchers, and researchers were the instruments since this is a qualitative study.

### 2.6 Data Quality/Trustworthiness

The trustworthiness of the study was determined by the principles of credibility, transferability, dependability, and conformability. Credibility refers to the confidence in the truth of the data. It is achieved through methods such as prolonged engagement with participants, triangulation of data sources and methods, and member checking, where participants review and validate the findings. Transferability relates to the extent to which findings can be applied to other contexts. This is supported by providing thick, detailed descriptions of the research setting and participants, allowing others to assess whether the results are relevant to their own situations. Dependability addresses the stability and consistency of the research process over time. It is ensured by maintaining an audit trail that documents all decisions, procedures, and changes throughout the study. Finally, conformability ensures that the findings are shaped by the participants’ responses and not researcher bias. This is supported through reflexivity, audit trails, and triangulation. Together, these components strengthen the overall trustworthiness of qualitative research and help ensure that the study is both meaningful and ethically sound. In this phenomenological research, the researcher explicitly involving themselves in data collection and management roles, minimizing potential biases, and the steps taken to mitigate them are crucial for ensuring the credibility and rigor of the study. Including strategies like reflexivity, bracketing, or peer debriefing helps to minimize the impact of personal assumptions and enhances the trustworthiness of the findings that were done. We incorporated this information to strengthen the methodological integrity of phenomenological studies and support the authenticity of participants’ lived experiences as presented in the research.

### 2.7 Role of Researchers and Coders

All interviews were transcribed verbatim and translated into English for analysis. Data were analyzed using thematic analysis consistent with phenomenological principles. Two independent researchers initially read the transcripts multiple times to familiarize themselves with the data and identify meaningful units. Each researcher independently generated initial codes, after which the coding results were compared and discussed to reach consensus. Discrepancies were resolved through discussion among the research team to ensure analytical rigor and reduce potential bias. Emerging codes were then grouped into categories and themes that reflected mothers’ lived experiences and challenges in caring for preterm infants in the NICU.

Data Processing and Analysis: Inductive thematic analysis was employed to characterize the interview data. It is a qualitative method used to identify patterns and themes that emerge directly from the data, without relying on pre‐existing theories or frameworks. The process began with familiarization, where researchers immerse themselves in the data by reading and re‐reading transcripts to gain a deep understanding of participants’ experiences. The researcher and one note taker were responsible for gathering and managing the interpreted data. Verbatim transcription was done by listening to audio record material, and the translated data was checked for similarity and consistency of meaning. This is followed by coding, where meaningful segments of text were labeled with descriptive or interpretive codes. These initial codes form the basis of a coding framework, which is developed iteratively as new insights arise. Researchers then search for patterns by grouping related codes into broader categories, forming preliminary themes that represent recurring concepts or ideas in the data. Once candidate themes were identified, we reviewed and refined to ensure they accurately reflect the data and are distinct from one another. This involves checking that each theme is well‐supported by data extracts and aligns with the research questions. Themes are then clearly defined, named, and organized into a coherent structure, often with sub‐themes. These steps collectively ensure that the thematic analysis is rigorous, grounded in the data, and reflective of participants’ lived experiences. Coding was conducted by reading and re‐reading the compiled transcripts using ATLAS.ti version 8 software. The authors also used the Consolidated Criteria For Reporting Qualitative Research (COREQ) is a 32‐item checklist to improve quality, transparency, and completeness qualitative research (Table 1). Themes were formed by connecting similar categories created by reading quotes, ensuring consistency of concepts and ideas. A report was written after checking coding data consistency.

**Table 1:** Consolidated criteria for reporting qualitative Studies (COREQ): 32‐Item Checklist.

No. item	Guide questions/Description	Reported on Page number
Domain 1: Research team and reflexivity		
Personal Characteristics		
1. Interviewer/facilitator	Which author/s conducted the interview or focus group?	1
2. Credentials	What were the researcher's credentials? E.g. PhD, MD	1
3. Occupation	What was their occupation at the time of the study?	1
4. Gender	Was the researcher male or female?	1
5. Experience and training	What experience or training did the researcher have?	1
Relationship with participants		
6. Relationship established	Was a relationship established prior to study commencement?	6
7. Participant knowledge of the interviewer	What did the participants know about the researcher? e.g. personal goals, reasons for doing the research	7
8. Interviewer characteristics	What characteristics were reported about the interviewer/facilitator? e.g. Bias, assumptions, reasons and interests in the research topic	7
Domain 2: study design		
Theoretical framework		
9. Methodological orientation and Theory	What methodological orientation was stated to underpin the study? e.g. grounded theory, discourse analysis, ethnography, content analysis	6–7
Participant selection		
10. Sampling	How were participants selected? e.g. purposive, convenience, consecutive, snowball	6–7
11. Method of approach	How were participants approached? e.g. face‐to‐face, telephone, mail, email	6–7
12. Sample size	How many participants were in the study?	6–7
13. Non‐participation	How many people refused to participate or dropped out? Reasons?	6–7
Setting		
14. Setting of data collection	Where was the data collected? e.g. home, clinic, workplace	7–9
15. Presence of non‐participants	Was anyone else present besides the participants and researchers?	7–9
16. Description of sample	What are the important characteristics of the sample? e.g. demographic data, date	7–9
Data collection		7–9
17. Interview guide	Were questions, prompts, guides provided by the authors? Was it pilot tested?	7–9
18. Repeat interviews	Were repeat interviews carried out? If yes, how many?	7–9
19. Audio/visual recording	Did the research use audio or visual recording to collect the data?	7–9
20. Field notes	Were field notes made during and/or after the interview or focus group?	7–9
21. Duration	What was the duration of the interviews or focus group?	7–9
22. Data saturation	Was data saturation discussed?	7–9
23. Transcripts returned	Were transcripts returned to participants for comment and/or correction?	7–9
Domain 3: analysis and findings		
*Data analysis*		9–10
24. Number of data coders	How many data coders coded the data?	9–10
25. Description of the coding tree	Did authors provide a description of the coding tree?	9–10
26. Derivation of themes	Were themes identified in advance or derived from the data?	9–10
27. Software	What software, if applicable, was used to manage the data?	9–10
28. Participant checking	Did participants provide feedback on the findings?	9–10
*Reporting*		
29. Quotations presented	Were participant quotations presented to illustrate the themes/findings? Was each quotation identified? e.g. participant number	11–25
30. Data and findings consistent	Was there consistency between the data presented and the findings?	11–25
31. Clarity of major themes	Were major themes clearly presented in the findings?	11–25
32. Clarity of minor themes	Is there a description of diverse cases or a discussion of minor themes?	11–25

### 2.8 Ethical Clearance and Consent to Participate

Ethical approval for the study was obtained from the Institutional Review Board (IRB) of Wachemo University under the reference number Ref No./WCU/SGS/552/2016. Permission to conduct the study was also obtained from the administration of the Hospital on September 20, 2023. Before participation, all eligible mothers were informed about the purpose of the study, procedures, potential benefits, and their rights as participants, including the right to decline or withdraw from the study at any time without affecting the care provided to them or their infants. Written informed consent was obtained from each participant prior to the interview. To ensure confidentiality, participants’ names and personal identifiers were not recorded, and audio files and transcripts were stored securely and accessible only to the research team.

## 3 Results

### 3.1 Socio‐Demographic Characteristics of the Participants

In this study, thirteen mothers of premature infants admitted to the NICU participated in an in‐depth interview. The mothers were between 24 and 36 years old. Regarding educational status, one mother had no formal education, four had primary school education, four had secondary school education, and four had a diploma (Table 2).

**Table 2:** Socio‐demographic characteristics of interviewed mothers in Wachemo University Nigist Elleni Mohammed Memorial Comprehensive Specialized Hospital, Hossana, Central Ethiopia 2024.

Participant ID	Age	Newborn age at birth (Weeks)	Time spent in the NICU (days)	Educational status	Ethnicity	Residence	Marital status	Occupational status	Religion	Parity	Mode of delivery	Type of pregnancy
P1	24	34	12	Primary Education	Silite	Rural	Married	Housewife	Muslim	Primiparous	C/s	Twins
P2	25	31	15	Diploma	Hadiya	Urban	Married	Government employee	Orthodox	Multiparous	SVD	Twins
P3	30	32	21	No formal education	Gurage	Rural	Married	Housewife	Muslim	Multiparous	C/s	Singleton
P4	29	34	9	Primary Education	Gurage	Urban	Married	Housewife	Muslim	Multiparous	C/s	Singleton
P5	36	32	16	Diploma	Hadiya	Urban	Married	Housewife	Protestant	Primiparous	SVD	Twins
P6	25	31	21	Secondary	Amhara	Urban	Married	Merchant	Orthodox	Primiparous	C/s	Singleton
P7	27	34	16	Secondary	Hadiya	Urban	Married	Merchant	Protestant	Primiparous	SVD	Singleton
P8	27	34	21	Diploma	Hadiya	Urban	Married	Government employee	Protestant	Multiparous	C/s	Singleton
P9	25	34	12	Diploma	Hadiya	Urban	Married	Government employee	Orthodox	Multiparous	SVD	Singleton
P10	30	32	8	Primary Education	Wolaita	Urban	Married	Housewife	Protestant	Multiparous	SVD	Singleton
P11	27	34	16	Secondary	Hadiya	Urban	Married	Merchant	Protestant	Primiparous	SVD	Singleton
P12	26	34	14	Secondary	Hadiya	Urban	Married	Housewife	Protestant	Primiparous	SVD	Singleton
P13	28	31	12	Primary Education	Hadiya	Rural	Married	Housewife	Protestant	Primiparous	SVD	Singleton

Abbreviations: C/s, cesarean section; SVD, spontaneous vaginal delivery.

### 3.2 Themes

Themes include the emotional experiences of mothers, mothers’ expectations, the needs of healthcare professionals, facility‐related factors, and perceived incompetence in taking care of preterm infants (Table 3).

**Table 3:** Summary of main themes and sub‐themes of mother experiences in Wachemo University Nigist Elleni Mohammed Memorial Comprehensive Specialized Hospital, 2024.

Main themes	Sub‐themes
Emotional experiences of mothers	Sadness and guilt
	Being shocked, worried, and anxious mothers
Mothers expectations	Mothers’ requirements of neonatal intensive care units in the hospitals
	Compassionate and respectful care
Needs of healthcare professionals	Health professionals related factors
	Security guards related factors
Health facility related factors	Resources
	Unavailability of medicines and laboratory reagents
Perceived incompetence in taking care of preterm infants	Lack of self‐confidence
	Perceived fear of taking care of a newborn

#### 3.2.1 Main Theme 1: Emotional Experiences of Mothers

In the neonatal intensive care unit (NICU), mothers went through strong and overwhelming emotions about their preterm babies. Their thoughts and feelings were so closely connected that it was hard to tell them apart. Seeing their babies surrounded by machines in such a critical condition was shocking and deeply upsetting. Mothers reported a wide range of emotions, including anger, fear, guilt, sadness, anxiety, frustration, and even moments of happiness. Many also felt depressed, out of control, and dissatisfied. These emotional experiences played an important role in the decisions mothers had to make while their babies were in the NICU.

##### 3.2.1.1 Subtheme 1.1: Sadness and Guilt

Most mothers in this study feel remorse and guilt for not having prepared for the possibility of a premature baby. Participants also reported that they could not see their newborn lying on the ground in the hospital room. They wanted to touch their baby and hear their baby's voice after enduring the pain of childbirth. Regarding admission to the NICU, the study participants mentioned feelings of guilt. They think they made a mistake, or that it happened because of the mother's problem, and then shorten to feelings of guilt and self‐blame. Some of the respondents mentioned that they were wrong about getting married early. Moreover, the manifested feeling of guilt and regret led to self‐isolation, the fear of disgrace, and suicidal ideation in the setting of infant death.

A mother said, “I felt a little guilty about giving birth early; that's why I didn't want a premature birth to occur.” (p4) Another mother said, “You told me whether the child survives or not, we will do the work to save you”. “I also thought, if my child doesn't survive, why should I get hurt? Afterwards, I was very stressed for 2 days. I also got tired.” (p8). Also, another mother said: “I just felt sad. My baby was so small, and my child was so innocent. I couldn't see her or hear her voice yet.” (p5).

In addition, the phenomenon of negative emotions was closely related to the impact of neonatal stays in NICUs. The long separation of the baby and the mother resulted in sadness and guilt. Another issue is when a relative comes to visit; she's afraid to tell the situation because she doesn't know exactly what's going on in the NICU.

##### 3.2.1.2 Subtheme 1.2: Being Shocked, Worried, and Anxious Mothers

Mothers described feeling shocked, worried, and anxious when their premature babies were admitted to the neonatal intensive care unit. Not knowing what the next step was and when discharge would be was a major concern for the mother. Due to the fact that the mother was not advised and educated on the general procedure of the newborn intensive care unit. Mothers also want to hear every minute about their babies’ health status. *“I was worried of what would happen next, what the child would become.” (*p3) *“Then I asked them about her. They said to pray for her and ask God to be with her. She was in the NICU at the time. I was really shocked.”* (p5). Another mother shared her story and said: *“I was wounded and lost my son. I was very worried and asked my husband what was wrong to my son. He said, your son has been put in a warm room.” (p8)* Another mother said*: “my husband was always very worried.” Even 1 day he complained and said, “Lord, what's wrong with him, me?” (p9)* Another mother shared her deep feelings about her first visit to the NICU: “*Actually, I'm afraid I'm gone for a baby, and I don't know if it will live or die. I'm so stressed, I'm so anxious, I'm so stressed that I'm getting a nervous breakdown*.” (p10).

#### 3.2.2 Main Theme 2: Mothers Expectations

##### 3.2.2.1 Subtheme 2.1: Mothers’ Requirements of Neonatal Intensive Care Units in the Hospitals

Mothers described their specific expectations of the healthcare team, particularly the nurses who cared for their infants 24 h a day. The study participants mentioned they expect nurses to relay information on laboratory data, screening, and testing. Furthermore, the mother wanted to know who is in charge of her baby and what medication was administered. The mothers with preterm newborns in hospitals expressed their needs in the following terms:

When I visited my child in the neonatal unit after the cesarean section, I expected the nurses to help me. I expected them to answer my questions, give me the details on my son, and let me see him. I wanted them to have a humanistic, compassionate attitude, especially as I was afraid of losing my son.

(p6)

There was a gap between mothers’ expectations and what was actually provided regarding the support that the health team, particularly nursing staff, was expected to provide. The mothers who did not get what they expected from the healthcare team were the same mothers who felt they were not supported by that team.

The health of professionals, especially nurses, did not support me at all. They were careless and didn't tell the truth. My husband and family were my main sources of support

(p9)

##### 3.2.2.2 Subthemes 2.2: Compassionate and Respectful Care

Some mothers stated that healthcare professionals provide compassionate and respectful care to mothers and parents during hospitalization. The mother described that: *“One night I went to the toilet and it really scared me. When I returned, one of the health professionals asked me what had happened. When I told him about my condition, he gave us a key to the staff toilet.” (p8)* Another mother said: *“I cried and refused to enter the ward again when I saw how fast his breathing and chest were moving, but the nurse insisted I go back. She told me he was getting better.” (p3)*.

#### 3.2.3 Main Theme 3: Needs of Healthcare Professionals and Security Guards

##### 3.2.3.1 Subtheme 3.1: Health Professionals‐Related Factors

Some mothers explained that healthcare providers do not respect mothers and parents during hospital stays. *“Not all health professionals lack compassionated and respectful care for their patients. However, some of them disrespect and misbehave on the mothers. Healthcare providers have several gaps; for example, they cannot understand other people's problems and are careless, causing harm to my baby and during labor, the midwife teased me” (p7)*. Also another mother who supported this idea said, *“The healthcare provider stayed on the cell phone for a long time and was really joking.” (p13)* Another mother also revealed that*: “They do not consider us human; they undermine, but I do not say these are the problems of all health professionals”. I am sorry I said that! (p4)*. One mother who participated in this interview said: *“When we come to see our children at night, health professionals are not always there, especially after midnight, unless there is a new admitting baby.” (p2)*.

##### 3.2.3.2 Subtheme 3.2: Security Guards‐Related Factors

One of the major challenges they faced in the ward was inconsistent rules from the security staff. Some mothers said security staff did not respect mothers and parents during hospitalization. And most mothers described that the time to visit their child was limited. Mothers wanted to see their babies, but the security guards did not permit this. The negative experiences, including verbal hostility towards the parents by security guards, led to feelings of hate towards the health facility. *“I hugged myself when I came in or went in. Sometimes it was security guards who prevented me from getting in.” (p2)* Another mother supports this idea and said*: “ones up on a time, I want to visit my baby but security guard comes to me and pushed me away. Imagine I have caesarian section operation patients.”(p6)*.

#### 3.2.4 Main Theme 4: Health Facility‐Related Factor

##### 3.2.4.1 Subtheme 4.1: Resources

This study showed that mothers had difficulty obtaining water due to a lack of adequate water and hygiene, and a lack of places to rest with their families. Lack of rest/sleeping space for mothers and parents is cited as a barrier during their hospital stay. A mother said, *“My husband doesn't sleep when he is so tired that he sits outside on the porch chair sleeping.” (p8)* Another mother said, *“My husband had a physical health problem because he did not have adequate space to rest or sleep during those 2 weeks in the NICU.” (p3)*.

Additionally, the lack of shower/bathroom facilities was cited as a barrier for mothers during their hospital stay; *“I can do it when I take the shower, but kids can't do it. Right now my newborn daughter has white spots on her armpits because I think she hasn't washed.” (p3)* Another mother said: *“When my father‐in‐law came, I was too embarrassed to approach him and greet him because my body smelled bad.” (p6)*.

##### 3.2.4.2 Subtheme 4.2: Unavailability of Medicines and Laboratory Tests

Almost all mothers reported a lack of medication and laboratory tests in the hospital. Due to the lack of medicines and laboratory tests for the care of the child, they are forced to purchase medicines in a private pharmacy outside the hospital and laboratory tests in a private clinic at high costs. This situation overwhelmed them and jeopardized their financial potential. One of the mothers said, “*It is very difficult because there is a lack of medication. In fact, the last time my husband went shopping outside the hospital and demanded five hundred birr, I didn't have the money on time, so we were much stressed.”* (p6).

Another respondent said: *“Mothers received free treatment at the hospital. However, since everything is now bought from private pharmacy, we no longer get gloves, medicines or other supplies.” (P13)* In addition, other mothers described their experiences with the unavailability of medications and materials needed to care for the child in the hospital in the following ways*. “This is just the help of the healthcare provider, nothing else. Gloves were also purchased from outside. That's another problem… Look, there are medicines that they send us to buy from outside (private pharmacy), and there are other things, so it would be nice if we could get them here, at least with the additional money. For example, they send us out at night to carry out laboratory tests. How do we get out tonight? It's scary.” (P7)*.

Another mother said, *“I am the victim of all the mothers who sleep here; I am the reason, and no one asks me to buy medicine. I do not have enough money. One of the professionals asked me to get a copy of your ID card. Then she told me that there was money to give. The woman next to me, without ID, gave me a copy of her ad. When she gave me one thousand five hundred birrs, I bought medicine from him.” (p. 3)*.

#### 3.2.5 Main Theme 5: Perceived Incompetence in Taking Care of Preterm Infants

Due to the separation and lack of involvement in the care of their infants, it was challenging for the mothers to develop their mothering skills and confidence. Over time, many mothers began to doubt their ability to care for their infants.

##### 3.2.5.1 Subtheme 5.1: Lack of Self‐Confidence and Fear of Taking Care of a Newborn

A premature birth poses major challenges for mothers, especially first‐time mothers. Mothers were unable to gain experience caring for their babies or learn to recognize their signals and needs because they were not involved in newborn care during their hospital stay. As a result, mothers felt less confident and more unable to fulfill their maternal roles.

I slept in the basement for 3 days, and after 3 days I saw my son, who had a tube in his nose. The room was full of heat, and I thought, Lord, you can do anything and then save my son, and I prayed that you would pay attention to my pain.” (p5) “Because he was premature, I didn't have enough confidence to take good care of him.

(p1)

Mothers felt they had a lot to learn before their children were discharged, such as feeding and changing diapers, but did not receive adequate guidance from nurses. As a result, these mothers were unsure whether they were adequately prepared to recognize the unique symptoms of premature babies.

My baby may be different from other babies. I had no idea whether I could take responsibility for her daily care, such as feeding her. Besides, I was also worried about how to care for her in other aspects.

(p2)

I was very afraid that my son would join me with other babies, so when I asked my husband if it was okay, the professionals in the care provider told me that they would put plasters on their feet. Also, I know the enclosure I bought, as my son said. I calmed down a bit after that.

(p6)

Another mother said that when one of the health professionals called my name and asked me if she wanted your daughter? I cried. I thought it was her. Day after day, I became so good that the health professionals told me that if all mothers were like us.

(p3)

## 4 Discussion

The study was conducted on the experience of neonatal intensive care units in mothers who had given birth to premature babies. It identified key themes such as emotional experiences, expectations, and mothers’ need from health professionals, healthcare‐related factors, and perceived lack of care. The mothers in the study experienced negative emotions such as shock, concern, and anxiety when their babies were born prematurely and admitted to the NICU. They were also worried when their children died. This is in line with previous studies in Ethiopia [25, 26] and elsewhere, where mothers reported that they experienced negative emotional feelings when their newborns were admitted to the neonatal intensive care unit due to the sight and sound of the various tubes and wires attached to the infants in the incubators [27, 28, 29, 30, 31, 32, 33].

Moreover, mothers expressed concern that the child who was born prematurely might not survive. This finding is supported by other studies conducted in China, which have shown that maternal anxiety is linked to how serious the newborn's condition is perceived to be and to uncertainty regarding the infant's chances of survival [34]. In Rwanda, mothers were traumatized by the belief that their babies would not survive [35]. This finding is also in line with a study carried out in Botswana, which found that participants were reluctant to believe that a small, fragile, and vulnerable baby would survive, which makes investment in a new relationship even more difficult [36].

Another important finding is that good support from the NICU team, especially nurses, is crucial to meet the expectations of the mother. Some mothers appreciated the role of the healthcare team in discussing their child's condition, encouraging mothers to visit their child, and helping them in their care. Furthermore, the mothers expressed appreciation for the emotional, psychological, and spiritual support offered by the sisters. However, some reported negative interactions with NICU staff and felt that the care provided lacked thoroughness. A few mothers mentioned that, despite seeking help from paramedics, they did not receive any emotional or psychological support. In addition, many mothers waited for nurses or other medical staff to communicate updates about their infants’ condition. This finding is consistent with the study conducted in Jordan [37].

The study shows that some mothers mentioned that the lack of compassionate and respectful care, and the lack of support of the healthcare providers and the security guards had negative consequences for the mothers, which meant that the healthcare providers and the security guards were not disciplined and they were not committed and cooperative in providing care in the NICU. Other studies have also shown that healthcare providers were insufficient and negligent [36, 37].

The study found that mothers faced a number of problems during their hospital stay, including the lack of medicines and laboratory tests, as well as facilities problems such as lack of toilet facilities, poor hygiene, and lack of family rest rooms. As a result, they had to pay high prices for medicines and laboratory tests in private clinics, and had to rely on private pharmacies to care for their children. A study in Wolaita Sodo, Ethiopia, and Ghana has shown that adequate facilities and equipment for pre‐term neonatal care, such as medicines, water, toilets, toilets, rest areas, and visiting hours for neonates, are lacking. This situation is putting at risk the economic potential of these communities and also raising concerns about the financial aspects of care [12, 16, 27, 28, 30, 37, 38, 39].

The study found that, in line with previous research, mothers often lack confidence and skills to care for premature babies. It also found that emotional and professional support were mutually exclusive, which is consistent with a Chinese study [40]. Knowledge of and involvement in childcare is strongly linked to trust in the care of infants for several reasons: development of skills through hands‐on experience and training, empowerment through understanding of the needs of premature infants, bonding and attachment through care activities, communication with healthcare professionals to better understand, and emotional support from healthcare professionals, family members, and support groups. This comprehensive approach allows mothers to face the challenges of newborns with resilience and confidence.

As a strength, the qualitative study is that it offers a profound understanding of the experiences of mothers with premature infants in neonatal intensive care units. Additionally, the emergence of anomalies beyond the established quantitative factors offers valuable direction for future research. However, a limitation of this study is its lack of generalizability due to its qualitative nature. Another limitation of the study is that interviews were conducted adjacent to a morning session, which may have influenced participants’ comfort, privacy, or emotional state during data collection, potentially affecting the depth and openness of their responses.

## 5 Conclusion

In conclusion, mothers whose premature babies have been admitted to the neonatal intensive care unit face emotional problems such as anger, fear, guilt, crying, anxiety, sadness, frustration, dissatisfaction, disappointment, guilt, and lack of self‐control. In addition, mothers suffered from a lack of rest areas, a lack of water for toilets and hygiene, a lack of medicines, laboratory tests, and limited time to visit their newborns. It is essential that healthcare professionals provide training and support to help build trust in the care of premature babies. By recognizing and addressing these specific needs, health professionals can contribute to positive outcomes for both the mother and the child during the stay in the neonatal unit. Based on the findings of the study, hospital management should provide adequate waiting areas, space for the care of newborns, avoid shortages of medicinal products and laboratory equipment, and provide a standardized NICU environment for both the hospital staff and the mothers to manage their hygiene and medical education, in order to minimize the risk of an emotional experience for the mothers.

## Author Contributions

**Bisrat Feleke Bubamo:** conceptualization, methodology, software, data curation, investigation, validation, formal analysis, visualization, project administration, resources, writing – original draft, writing – review and editing. **Gizachew Beykaso, Genanew Kassie Getahun, Markos Selamu:** conceptualization, methodology, data curation, investigation, validation, formal analysis, supervision, funding acquisition, visualization, project administration, resources, writing – original draft, writing – review and editing. **Hamdela Tumiso:** conceptualization, methodology, software, data curation, investigation, validation, funding acquisition, visualization, project administration, writing – review and editing, writing – original draft. **Eyob Ketema Bogale:** conceptualization, methodology, software,data curation, investigation, validation, formal analysis, supervision, funding acquisition, visualization, project administration, resources, writing – original draft, writing – review and editing. **Feleke Doyore Agide:** conceptualization, methodology, software, data curation, investigation, validation, formal analysis, supervision, funding acquisition, writing – review and editing, writing – original draft, resources, project administration, visualization. **Melaku Eriso:** conceptualization, methodology, data curation, investigation, validation, formal analysis, supervision, funding acquisition, visualization, project administration, resources, writing – original draft, writing – review and editing, conceived and designed the study.

## Funding

The authors have nothing to report.

## Conflicts of Interest

The authors declare no conflicts of interest.

## Transparency Statement

The lead author Feleke Doyore Agide affirms that this manuscript is an honest, accurate, and transparent account of the study being reported; that no important aspects of the study have been omitted; and that any discrepancies from the study as planned (and, if relevant, registered) have been explained.

## Data Availability

The data analyzed during the current study are available from the corresponding author on a reasonable request.
